# First Molecular Epidemiological Study of Cutaneous Leishmaniasis in Libya

**DOI:** 10.1371/journal.pntd.0001700

**Published:** 2012-06-19

**Authors:** Ahmad Amro, Aisha Gashout, Hamida Al-Dwibe, Mohammad Zahangir Alam, Badereddin Annajar, Omar Hamarsheh, Hend Shubar, Gabriele Schönian

**Affiliations:** 1 Faculty of Pharmacy, Al-Quds University, Abu-Dies, Jerusalem, Palestine; 2 Libyan National Centre for Infectious Diseases and Control, Tripoli, Libya; 3 Department of Parasitology, Faculty of Veterinary Science, Bangladesh Agricultural University, Mymensingh, Bangladesh; 4 Institut für Mikrobiologie und Hygiene, Charité Universitätsmedizin Berlin, Berlin, Germany; 5 Department of Biological Sciences, Faculty of Science and Technology, Al-Quds University, Jerusalem, Palestine; Ege University, Turkey

## Abstract

**Background:**

Cutaneous leishmaniasis (CL) is a major public health problem in Libya. The objective of this study was to investigate, for the first time, epidemiological features of CL outbreaks in Libya including molecular identification of parasites, the geographical distribution of cases and possible scenarios of parasite transmission.

**Methodology/Principal Findings:**

We studied 450 patients that came from 49 areas distributed in 12 districts in north-west Libya. The patients' ages ranged from 9 months to 87 years (median age 25 years); 54% of the cases were males. Skin scrapings spotted on glass slides were collected for molecular identification of causative agent. The ribosomal internal transcribed spacer 1 (ITS1) was amplified and subsequently characterized by restriction fragment length polymorphism (RFLP) analysis. In total, 195 samples were successfully identified of which 148 (75.9%) were *Leishmania major*, and 47 (24.1%) *Leishmania tropica*. CL cases infected with *L. major* were found in all CL areas whereas *L. tropica* cases came mainly from Al Jabal Al Gharbi (46.4%), Misrata (17.8%) and Tarhuna districts (10.7%). A trend of seasonality was noticed for the infections with *L. major* which showed a clear peak between November and January, but was less pronounced for infections by *L. tropica*.

**Conclusion:**

The first molecular study on CL in Libya revealed that the disease is caused by *L. major* and *L. tropica* and the epidemiological patterns in the different foci were the same as in other Mediterranean foci of CL.

## Author Summary

Cutaneous leishmaniasis (CL) is caused by protozoan parasites of the genus *Leishmania*. The disease is characterized by the formation of chronic skin lesions followed by permanent scars and deformation of the infected area. It is distributed in many tropical and subtropical countries with more than 2 million cases every year. During the past few years CL has emerged as a major public health problem in Libya. So far, diagnosis was based on clinical symptoms and microscopic observation of parasites. Disease outbreaks were not investigated and the causative leishmanial species of CL were not identified so far. Our study indicates the presence of two coexisting species: *Leishmania major* and *Leishmania tropica*. These results are crucial in order to provide accurate treatment, precise prognosis and appropriate public health control measures. The recent armed conflict in Libya that ended with the Gadhafi regime collapse on October 2011 has affected all aspects of the life in the country. In this study we discussed multiple risk factors that could be associated with this conflict and present major challenges that should be considered by local and national health authorities for evaluating the CL burden and highlighting priority actions for disease control.

## Introduction

Leishmaniasis is still one of the world's most neglected diseases infecting some 2 million humans each year in more than 98 countries or territories. It occurs in three clinical forms including cutaneous leishmaniasishttp://hinari-gw.who.int/whalecomwww.sciencedirect.com/whalecom0/science/article/pii/S1201971211000877 - hit41 (CL), mucocutaneous leishmaniasis (MCL), and visceral leishmaniasis (VL). CL is the most common form of the disease in North African countries where three *Leishmania* species are considered as causative agents; *Leishmania major*, *L. tropica* and less frequently *L. infantum* [1], [2], [3], [4]. *L. tropica* is considered as species complex including *L. killicki* [5], for which a separate species status was not supported by different molecular analysis [6], [7], [8]. In terms of its infection cycle, two types of CL are commonly found; zoonotic cutaneous leishmaniasis (ZCL) caused by *L. major* and *L. infantum*, and anthroponotic cutaneous leishmaniasis (ACL) caused by *L. tropica* in urban areas [9]. Zoonotic transmission of *L. tropica* has been however, documented for Moroccan, Israeli and Palestinian CL foci and dogs and hyraxes have been incriminated as putative reservoir hosts of the parasite [10], [11]. The principal reservoirs of *L. major* in North Africa are the fat sand rat *Psammomys obesus* and several *Meriones* species, while canids are the reservoir for *L. infantum* [9].

In the Old World *Leishmania* parasites are transmitted by female sand flies belonging to different species of the genus *Phlebotomus* ((Diptera: Psychodidae). In the Mediterranean Basin, *Phlebotomus papatasi* is the main proven vector of *L. major* and *P. sergenti* that of *L. tropica*. However, other vectors for *L. tropica* were described such as *P. guggisbergi* in Kenya [12] and *P. arabicus* in Tiberias [13]. *L. infantum* is transmitted by different species of the subgenus *P. Larroussius* as reviewed elsewhere [14], e.g. by *P. perfiliewi*, *P. perniciosus* and *P. longicuspis* in Tunisia [15] and Algeria [6], and *P. ariasi*, *P. perniciosus* and *P. longicuspis* in Morocco [6].

The biting season of sand flies in the Mediterranean Basin extends from May to October [13], [16] after which a peak of infections is recorded until February of the next year. In Tunisia, seasonal occurrence of CL cases was described [15]. Two peaks of emerging cases in August–September and December are probably related to the seasonal activity of the respective phlebotomine sand fly vectors [17]. However, trend of seasonality of ZCL and ACL was noticed in some countries [18]; the maximum number of cases of ZCL is recorded in September and October and ACL peak is seen in March and April [18].

In Libya, CL is widespread in the north-western region. The first case of CL was reported in 1930, followed by recording of 40 cases in 1971 in Nalut near the Tunisian border [3], [19], [20]. In the following years several CL cases have been subsequently occurred in the west and south-west of Tripoli, Al-Badarna [21] and Yafran areas [3], [22]. The causative agents of CL in Libya have however, never been identified.

The diagnosis of CL in Libya is based on clinical signs of the disease and microscopic observation of parasites in stained skin biopsies [3], [22]. Specific and sensitive molecular diagnostic tools have not yet been implemented and information about disease distribution, parasite life cycle and combining risk factors is confined.

The objective of this study was to investigate epidemiological features of CL outbreaks in Libya. This includes the detection and molecular identification of causative *Leishmania* species, the geographical distribution of cases and indications for possible scenarios of parasite transmission and life cycle. To our knowledge, this is the first molecular epidemiological study of CL in Libya.

## Materials and Methods

### Sample collection and geographic distribution

**Figure 1:**
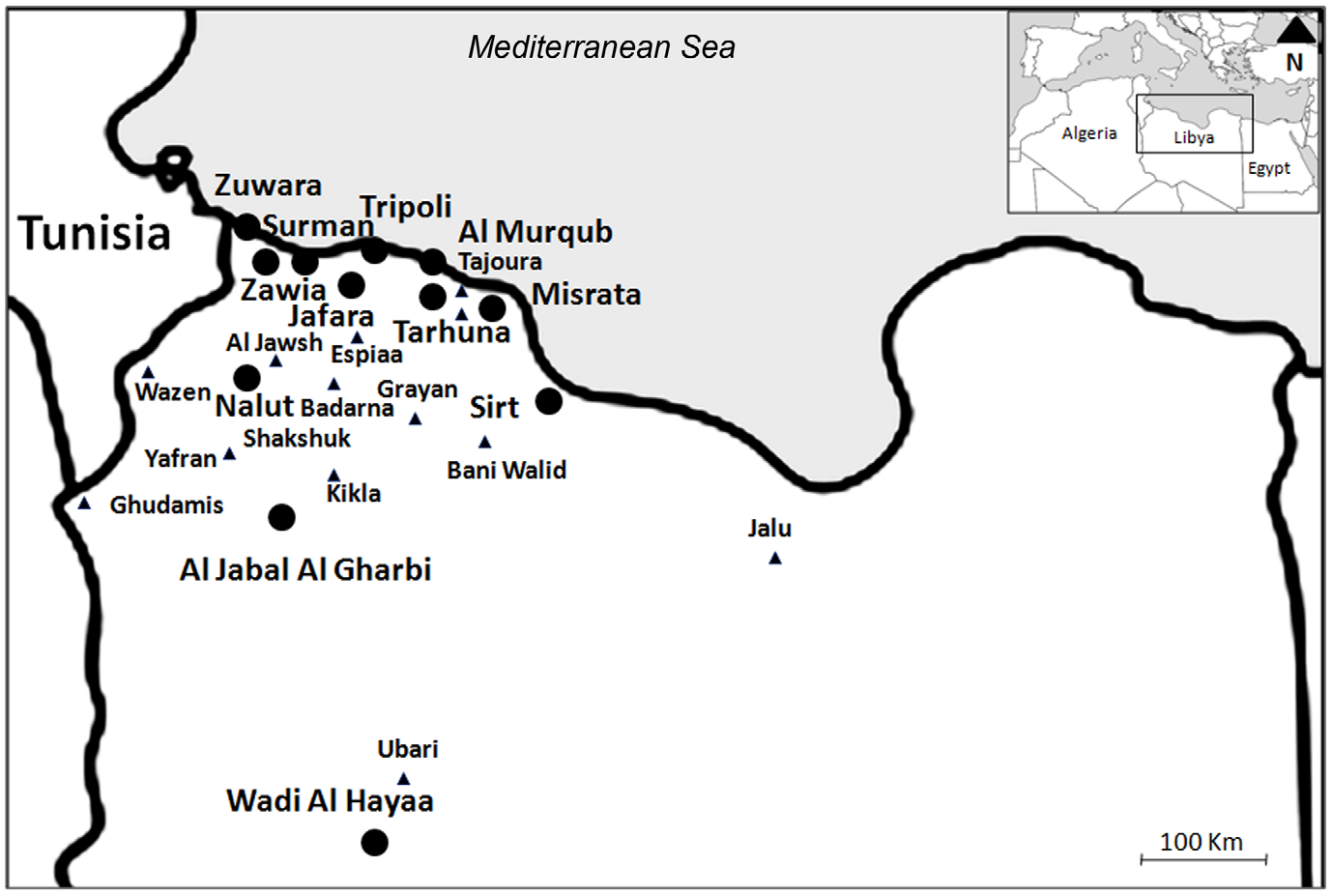
Geographical distribution of CL in Libya. The map of Libya showing the areas endemic for CL. •Districts. ▴Endemic areas.

Previously collected clinical specimens and patient's profiles were taken from the archive of the Libyan National Centre for Infectious Diseases and Control (LNCIDC). These specimens and patient's profiles have beenarchived since 1995 for a total of 450 patients who have been referred to hospitals with skin lesions typical for CL. These cases were confirmed as CL patients based on clinical symptoms and microscopic examination. The patients came from different areas endemic for CL in Libya (Fig. 1). According to ethical approval of this study, all samples were anonymized. Study design and procedures were revised and approved by the Libyan National Centre for Infectious Diseases and Control.

Patient's profiles including date of sampling, age, sex and location were collected for epidemiological analysis. According to LNCIDC procedures, specimens were taken from lesions and adjacent normal-looking skin around them. Skin was cleaned and sterilized with disinfectant. Tissue biopsies for making stained smears were taken using a disposable scalpel blade. A small incision was made in the cleaned margin of lesions with the point of the blade to remove and pick up skin tissue which was then smeared on clean glass microscope slides. These slides were stained with Wright's Giemsa and examined for the presence of amastigote bodies by light microscopy at 400×magnification. Positive slides were stored by LNCIDC. All slides and corresponding patients' profiles that were stored in LNCIDC archive since 1995, were made available for this study. Slides with missing patient's profiles were excluded. The selected slides were subjected to DNA extraction and molecular identification of the causative *Leishmania* species.

### DNA extraction

Unstained smears were kept at 4°C until DNA extraction. To each glass slide, 250 µl lysis buffer (50 mM NaCl, 50 mM Tris, 10 mM EDTA, pH 7.4, 1% v/v Triton X-100 and 100 µg of proteinase K per ml) were added. After dissolving, the solution was transferred to 1.5 ml reaction tube. Cell lysis was accomplished after incubation over night at 60°C. Lysates were then subjected to phenol–chloroform extraction as described elsewhere [23], [24]. A negative extraction control was used to control for possible contamination during DNA extraction process [25]. DNA purification, to reduce the amount of possible polymerase chain reaction (PCR) inhibitors, was done using the Nucleospin® Extract kit. A final volume of 30 µl obtained were kept at −20°C until used.

### Molecular characterization of parasites

A PCR- restriction fragment length polymorphism (RFLP) approach was applied for the detection and identification of the *Leishmania* parasites. The ribosomal internal transcribed spacer 1 (ITS1) was amplified using the primer pair L5.8S and LITSR [26]. Amplicons were analyzed on 1.5% agarose gels by electrophoresis and visualized by UV light. A reaction was considered positive when a band of the correct size (300 to 350 bp) was observed. A negative and positive control containing distilled water and DNA of *Leishmania turanica*, respectively, were included during PCR to ensure reliability, validity and to check for possible contaminations of the amplification reactions. In order to detect possible inhibitors, PCR inhibition control that contained both the sample DNA and DNA purified from cultured promastigotes of *L. turanica* was run along each sample.PCR product was digested with the restriction endonuclease Hae*III*. Produced fragments were separated by electrophoresis on 2.5% agarose gels and compared with those of WHO reference strains of *L. major* (MHOM/PS/2001/ISL659), *L. tropica* (MHOM/PS/2002/63JnF21) and *L. infantum* (MHOM/TN/1980/IPT1) as described elsewhere [24]. The three species co-exist in North African countries and cause CL [27], [28], [29].

## Results

The 450 patients investigated in this study came from 49 areas in 12 districts of north-west Libya. These districts from east to west and from north to south are; Sirt, Misrata, Al Murqub, Tarhuna, Tripoli, Jafara, Surman, Zawia, Zuwara, Nalut, Al Jabal Al Gharbi and Wadi Al Hayaa (Fig. 1).

The ribosomal ITS1 could be successfully amplified and characterized in DNA samples purified from 195 of the glass slides. The rest of the samples were either inhibited or failed to amplify.

**Figure 2:**
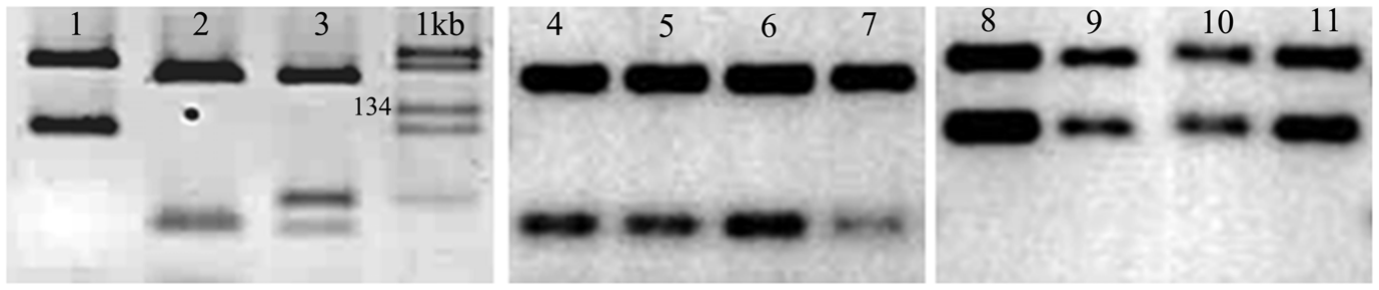
Molecular identification of causative CL species. Restriction fragment length polymorphism (RFLP) analysis of the amplified internal transcribed spacer 1 region (ITS1) digested with restriction enzyme *Hae*III and analysed by electrophoresis on 2.5% agarose gels. Three reference strains were used for comparison; Lane 1 = *L. major*: MHOM/PS/01/ISL659, Lane 2 = *L. tropica*: MHOM/PS/02/63JnF21 and Lane 3 = *L. infantum* MHOM/TN/1980/IPT1. 1 kb = molecular size marker. All other lanes show digested PCR product from clinical materials; lanes 4–7 = *L. tropica* cases from Al Jabal Al Gharbi, Misrata and Tarhuna districts; lanes 8–11 = *L.major* cases from Tripoli, Sirt, Misrata, Al Murqub.

ITS1-PCR produced a single amplicon of 300–350 bp which is characteristic for all medical relevant *Leishmania* species (data not shown). The digestion of the PCR product with endonuclease Hae*III* revealed that CL in Libya is caused by at least two *Leishmania* species. The RFLP profiles for 148 samples (75.9%) consisted of two bands (160 and 210 bp) and were identical to that of the *L. major* WHO reference strain, whereas 47 samples (24.1%) showed two bands (185 and 57 pb) as did the *L. tropica* WHO reference strain.(Fig. 2). The number of microscopically positive and of PCR positive slides, and the results of species identification are given per year for the total period 1995–2008 (Table 1).

**Table 1:** Species identification from positive slides and positive ITS1 PCR.

Year	+ve Slides	*L. major*	*L. tropica* (%)	Total spp*
**1995**	54	11	0	11
**1996**	5	0	0	0
**01997**	7	0	0	0
**1998**	30	1	3	4
**1999**	3	1	0	1
**2000**	5	0	0	0
**2001**	4	0	0	0
**2002**	6	0	2	2
**2003**	9	1	2	3
**2004**	43	19	5	24
**2005**	26	11	7	18
**2006**	63	22	9	31
**2007**	59	23	6	29
**2008**	136	59	13	72
**Total**	**450**	**148**	**47**	**195**

Number of microscopically and PCR positive slides as well as the results of *Leishmania* species identification per year given for the total period from 1995 to 2008.

CL cases infected with *L. major* were scattered in all districts. Most of the *L. tropica* cases came, however from Al Jabal Al Gharbi (46.4%), Misrata (17.8%) and Tarhuna districts (10.7%) (Fig. 1).

The age distribution at illness onset ranged from 9 months to 87 years (median age 25 years). No significant differences among age groups have been found. The male∶female ratio was 1.17∶1 (54% males). Cases were treated with i.m Sodium stibogluconate (Pentostam®) (10–20 mg/kg body weight) daily for 10–20 days and complete healing was observed in most cases. In poorly responding cases and when parenteral administration of Pentostam was not possible, patients were treated with either oral Rifampicin 1200 mg/day combined with 300 mg/day isoniazide for 6–8 weeks or received a cryo-therapy accompanied by intralesional Pentostam treatment.

**Figure 3:**
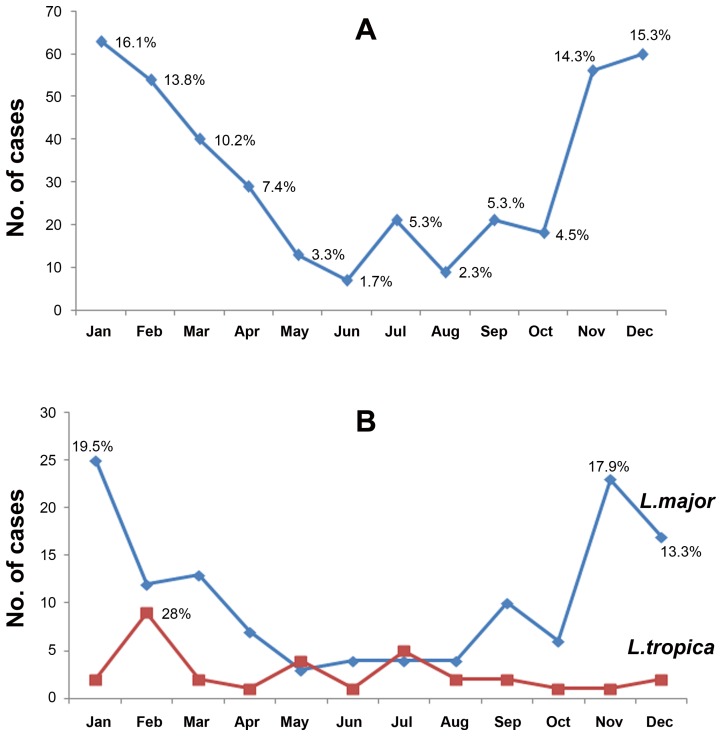
Seasonal distribution of CL in Libya. **A.** Seasonal distribution of CL cases as reported by the Libyan National Centre for Infectious Diseases and Control (1995–2008). The highest peak was from November till February. **B.** Seasonal distribution of CL cases caused by *L.major* showing a peak from November till January and by *L.tropica* that peaked in February. These results are based on data collected form 1995 to 2008.

The highest number of CL cases (60%) was recorded after the end of sandfly transmission season, during the months November-February with highest peak being in January (16.1%). The total number of cases declined in March (10.2%) and April (7.4%) and was lowest during the months May–October (Fig. 3A). It was noticed that 50.7% of CL caused by *L. major* occurred from November–January, while the highest peak of CL caused by *L. tropica* (28%) was in February (Fig. 3B).

## Discussion

In this study, *Leishmania* parasites causing CL in Libya have been detected in clinical specimen and identified at the species level by using a PCR-RFLP approach, and the geographical and demographic distribution of cases and the disease dynamics were investigated.

Of the 450 samples, 195 were successfully amplified and characterised by PCR-RFLP. The remaining specimens were possibly inhibited due to presence of impurities in the DNA extracts, which were not sufficiently eliminated during DNA purification process, or to degraded DNA. Moreover, previous reports have shown that ITS1 PCR-RFLP does not have an ideal sensitivity as diagnostic tool [30], [31]. Hence, for diagnostic purposes, PCR inhibition and extraction controls [25] should be included in every experiment to avoid false negative results. ITS1 PCR-RFLP revealed the presence of two co-existing species causing of CL in Libya; *L.major* and *L.tropica*. This result was not surprising since these two species are the most prevalent agents of CL around the Mediterranean Basin. *L. infantum* which has been identified as another causative agent of CL in other North African countries [28], [32] was not present in the specimens investigated in this study. Beside parasite DNA degradation and the presence of factors inhibiting PCR, the failure of *L. infantum* detection in this study sample might be related to the usually low parasite load in CL lesions caused by this species.

The clinical spectrum of CL is broad, lacks specificity, and may mimic that of other skin infections such as staphylococcal, streptococcal, mycobacterial ulcer and fungal infections. Microscopy does not allow for species differentiation and lacks sensitivity. Moreover, CL cases caused by *L. tropica* tend to last longer and are more difficult to treat than those caused by *L. major*, hence sensitive and species-specific diagnostic method should be mandatory at the primary health care level.

CL is affecting all age groups in Libya. The male∶ female ratio indicated that the infection rate among males was slightly higher than females (1.17∶1). The possible explanation is that men have a habit of sleeping outside their homes during hot nights where they may be more prone to get bitten by infected sand flies compared to women who tend to have fewer activities outside their homes. These results are in consistence with a previous study done by El-Buni *et al* [3]. Sodium stibogluconate (Pentostam) is the first line treatment of CL in Libya. Until recently, few resistant cases were reported; however, there was no systematic reporting of these cases by physicians. A better reporting system has to be established and resistant cases need to be investigated.

All CL cases in Libya were originated from the north-western districts of the country exclusively. These districts have typical Mediterranean costal climate in the upper northern districts like Tripoli, and semiarid and arid climate in Al Jabal Al Gharbi and Wadi Al Hayaa to the south. Like many other countries around the Mediterranean Sea, climatic and environmental conditions and development of agricultural activities in these districts may be favourable for transmission of *Leishmania* [4], [33]. Infection with *L. tropica* seems to be rather hypoendemic compared with *L. major* which was found in all endemic districts. This pattern of infections within these districts is essentially due to different life cycles of the two species that have to be investigated.

No studies were conducted on transmission cycles of CL in Libya. Zoonotic transmission has been shown for *L. major* in all areas of its distribution including the Middle East and North Africa where the parasites are transmitted by *P. papatasi* from its reservoir hosts *Ps. obesus* and *Meriones* spp to humans [34], [35], [36]. CL due to *L. tropica* is considered as an anthroponosis in many countries, especially in densely populated cities [37], [38], and transmitted via its natural vector *P. sergenti* between humans, nonetheless, zoonotic transmission of *L. tropica* was recently proven in the Middle East [10] and suggested to occur in less populated rural areas in the Middle East and North Africa. This study revealed a clear seasonality for the CL incidence in Libya that is mainly related to infections by *L. major* which show a peak during November-January. Infections with *L. tropica* seem to occur at relatively low level throughout the year with a small peak in February. Moreover, this seasonal distribution patterns may be caused by differences in the incubation period of *L. major* and *L. tropica* infections. The latter seems to be more insidious compared to *L. major* infection and to have a longer incubation period with fewer inflammatory lesions and a tendency toward chronic inflammation. These results are consistent with those reported from other CL foci in the Middle East and Central Asia [3]. However, the results are limited by the small number of samples that were available in the LNCIDC archive for the period 1995–2008. Table 1 shows that the number of collected slides and of PCR positive samples for which species identification were possible were not evenly distributed throughout the total period. Species identification was more achievable from samples collected after 2002 (Table 1). This can be due to the smaller number of samples collected earlier and possible degradation of DNA extracted from older slides.

A previous study has shown that *P. papatasi* is the most abundant sand fly species in Libya, followed by *P. sergenti* [3]. This might explain the abundance of *L. major* (75.9%) compared to *L. tropica* (24.1%) in our results. Wild rodents such as *Meriones libycus*, *M. shawi* and *Gerbillus gerbillus* have been found in the studied areas and considered as possible animal reservoirs for *L. major*. Parasites were however, not isolated from these putative vectors and reservoirs hosts in order to prove their role in parasite transmission. Thorough investigation of the density and distribution of sand fly vectors and putative animal reservoirs, both for infections by *L. major* and *L. tropica*, is needed for a better understanding of the epidemiology of CL in Libya and for implementing appropriate control measures.

In response to increasing number of CL cases during the last decades in Libya, a National Control Program (NCP) was launched in 2006 with the main objective to prevent the eruption of epidemics and to stop the spread of CL to so far non-endemic areas. Methods to control rodents were implemented and led to the elimination of 85% of the targeted population of rodents in endemic areas. A wide scale campaign of vector control was also applied utilizing fogging and residual spraying of pyrethroids. Hence the number of cases has progressively decreased from 7180 in 2006 to 1800 in 2008 (LNCIDC reports). The armed conflict which occurred in Libya from February till October 2011 has affected all aspects of the life in the country. Risk factors are known to accumulate during such conflict leading to an enhanced transmission of infectious diseases. In Libya, the primary health care services were interrupted or massively impaired in some of the endemic areas. This has resulted in poor detection and treatment of CL cases, inadequate surveillance and the complete interruption of the *Leishmania* national control program (NCP) in Libya. Massive human migration from cities to villages and camps took place from May to October, the main CL transmission season. This is an additional risk factor because people were more prone to get bitten and infected and, at the same time, had poor access to essential health services. The quantification and the containment of these risk factors are major challenges and should be considered by health policy makers and health professionals in order to evaluate the CL burden and to highlight priority actions for the disease control.

Since different Leishmania parasite species can cause CL in Libya, different reservoir hosts and insect vectors are involved in the transmission of CL and must be carefully determined before control measures are instituted. Understanding the different parasites' life cycles and parasite-vector-reservoir interplays is vital for applying effective prevention strategies, control measures, for the design of surveillance protocols or guidelines for monitoring the burden of CL in Libya and for evaluating the effectiveness of these control measures.
